# Ligament inter-vésico-rectal séparant les hémi-matrices d'un utérus bicorne unicervical - à propos d'un cas rare

**DOI:** 10.11604/pamj.2013.15.27.2773

**Published:** 2013-05-20

**Authors:** Sofia Jayi, My Abdelilah Melhouf

**Affiliations:** 1 Service de gynéco-obstétrique 2, CHU HASSAN II, Université Sidi Mohammed Benabdellah, Fès, Maroc

**Keywords:** Utérus bicorne, échographie pelvienne, malformation, métrorragie, uterus bicornis, pelvic ultrasound, malformation, metrorrhagia

## Image en médicine

Nous rapportons le cas d'une patiente âgée de 64 ans, ménopausée il y a 8 ans, grande multipare (toutes les grossesses menées à terme avec accouchement par voie basse), admise pour métrorragie post ménopausique avec douleurs pelviennes chroniques. L'examen clinique a trouvé une cloison vaginale longitudinale souple, un col aspiré et la taille de l'utérus était difficile à apprécier (patiente obèse). L'échographie pelvienne a objectivé la présence de 2 matrices avec signe de V vésical, un endomètre épaissi de 6.4mm, ce qui a permis en association avec les données cliniques, d'évoquer un utérus bicorne unicervical. Un complément par échographie rénale à la recherche d'éventuelle malformation associé s'est révélé sans particularité. Dans le but d'explorer la cavité utérine on a réalisé une hystéroscopie diagnostic qui n'a pu visualiser qu'une seule cavité tubulée avec impossibilité de progression; d'où la décision d'hystérectomie diagnostic. Au cours de cette dernière, l'exploration a confirmé la malformation type: utérus bicorne unicervical (2 hémimatrices (a-b) avec leurs annexes (étoiles), mais en outre, a mis en évidence une variété rare de cette entité qu'est l'association d'un ligament inter- vésico-rectal (c) étendu entre la vessie en avant (d) et la face antérieure du recto-sigmoide en arrière (e), séparant les 2 hémimatrices (A). L'hystérectomie a été réalisée avec libération du ligament inter-vésico-rectal avec délicatesse, de crainte de blesser l'éventuel diverticule vésicale pouvant parfois s'insinuer dans son épaisseur (B,f: portion cervico-isthmique). Par ailleurs l'étude histologique n'a pas trouvé d'anomalie sur l'endomètre.

**Figure 1:**
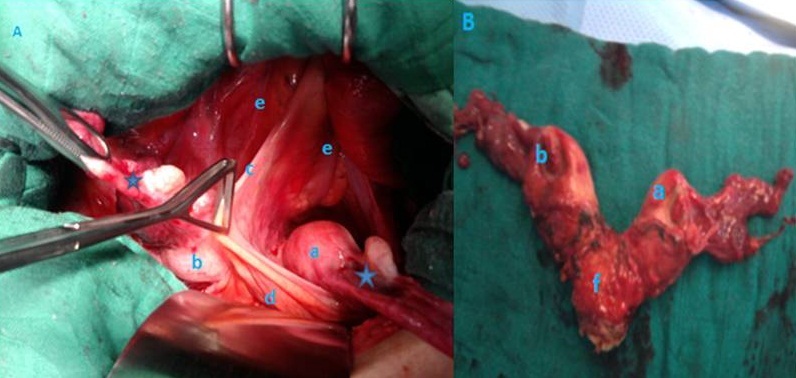
Malformation type: utérus bicorne unicervical (2 hémimatrices (a-b) avec leurs annexes (étoiles), mais en outre, a mis en évidence une variété rare de cette entité qu'est l'association d'un ligament inter- vésico-rectal (c) étendu entre la vessie en avant (d) et la face antérieure du recto-sigmoide en arrière (e), séparant les 2 hémimatrices

